# Prevalence of Trismus in Head and Neck Cancer Patients in Kerala, South India

**DOI:** 10.7759/cureus.75593

**Published:** 2024-12-12

**Authors:** Gopi B Krishna, Radhu Raj, Anju James, Nitin A Krishnan, Subramaniam Iyer, Anil Mathew, Chandrashekar Janakiram

**Affiliations:** 1 Public Health Dentistry, Amrita School of Dentistry, Amrita Vishwa Vidyapeetham, Kochi, IND; 2 Prosthodontics and Implantology, Amrita School of Dentistry, Amrita Vishwa Vidyapeetham, Kochi, IND; 3 Oral Medicine and Radiology, Amrita School of Dentistry, Amrita Vishwa Vidyapeetham, Kochi, IND; 4 Head and Neck Surgery, Amrita School of Dentistry, Amrita Vishwa Vidyapeetham, Kochi, IND

**Keywords:** head and neck cancer, oral cancer, prevalence, risk factors, trismus

## Abstract

Introduction: Trismus is a common complication of head and neck cancer (HNC) treatment. Understanding its prevalence and its risk factors is vital for enhancing clinical outcomes and the overall quality of life of these patients.

Objective: The study aimed to assess the prevalence and the factors associated with trismus among HNC patients.

Methods: A retrospective study was conducted at a tertiary hospital in Kerala, from January 2024 to April 2024. A total of 162 patient records were included in the study. Mouth opening ≤ 35 mm is considered trismus.

Results: Prevalence of trismus was 77 (46.9%). Gender, age group, smokeless tobacco use, tumor area, and bone involvement are the factors associated with the development of trismus (p < 0.05).

Conclusion: Nearly 50% of HNC patients develop trismus. Understanding the factors associated with trismus will aid in effectively managing patients and thereby improving patient outcomes.

## Introduction

Head and neck cancer (HNC) is a significant health concern, encompassing cancers occurring in the mouth, throat, nose, sinuses, and salivary glands [1]. These cancers can be aggressive and significantly impact the quality of life and survival rates. In India, HNC is particularly prevalent, accounting for 30%of allcancer cases [2]. The high incidence of HNC in India is attributed to various factors, including tobacco and alcohol use, betel quid chewing, and viral infections such as human papillomavirus (HPV) and Epstein-Barr virus (EBV) [3].

One of the key challenges in managing HNC is the high percentage of cases diagnosed at anadvanced stage(66.6%), especially in India, often due to a lack of awareness about early symptoms, limited access to healthcare facilities, and cultural and social factors [4]. Advanced-stage HNC is more difficult to treat and is associated with poorer outcomes compared to early-stage disease. The most common complications of HNC include hearing loss, difficulty eating, dental problems, thyroid issues, and difficulty breathing.

Trismus, or restricted mouth opening, is a common and debilitating complication of HNC treatment, resulting from factors such as tumor invasion into masticatory muscles, surgical trauma, and the effects of radiation therapy. Trismus is present in almost 17.3% ofHNC patients and significantly impacts a patient's quality of life, affecting their ability to eat, speak, and maintain oral hygiene [5]. The prevalence of trismus in treated HNC patients varies widely in the literature, ranging from 5% to 79%. It can also lead to psychological issues such as anxiety and depression, as patients may feel self-conscious about their appearance and ability to function normally [6].

Managing trismus in HNC patients requires a multidisciplinary approach, involving oral and maxillofacial surgeons, radiation oncologists, speech therapists, and physiotherapists. Treatment options include physical therapy, such as jaw exercises and stretching techniques, and the use of devices to improve mouth opening. In severe cases, surgical intervention may be necessary to release tight muscles and improve jaw mobility.

Early diagnosis is critical for planning treatment and prevention strategies for trismus, a condition characterized by restricted mouth opening. Various methods are employed to treat trismus, with physical therapy being a primary treatment method, often used alone or in conjunction with other treatments [7]. Physical therapy typically includes active range-of-motion exercises, hold-relax techniques, manual stretching, and joint distraction [8]. Post-surgery, patients are advised to undergo physiotherapy, which may include passive activities, the use of tongue blades, a Fergusson mouth gag, and Hiester’s jaw opening device [9].

Despite advancements in HNC treatment, trismus remains a significant and often persistent complication that significantly affects patients' quality of life. However, there is a lack of studies specifically focusing on the prevalence and factors associated with the trismus in the South Indian population. Understanding the prevalence of trismus, and its associated risk factors, is crucial for improving clinical outcomes and enhancing the quality of life of HNC patients. Therefore, this study aims to address this gap by assessing the prevalence of trismus in HNC patients in South India and identifying factors contributing to its development, with the goal of informing clinical practice and improving patient care.

## Materials and methods

A retrospective study was conducted at the Amrita Institute of Medical Sciences, Kerala, India, from January 2024 to April 2024. Medical records on the post-operative period of all patients aged 18 years or older who were at different stages of HNC rehabilitation were reviewed. A total of 162 patient records were included in the study. These records were selected based on the strict inclusion and exclusion criteria to ensure the reliability and relevance of the study outcomes.

Inclusion criteria encompassed patients who had received treatment for cancer affecting specific sites in the head and neck region. These sites included the tongue, floor of the mouth, cheek, lip, alveolar process of the mandible or maxilla, buccogingival vault of the mandible or maxilla, base of the tongue, tonsil, palate, lateral and dorsal walls of the oropharynx, pharyngeal arch, nasopharynx, vestibulum nasi, nasal cavity, nasal sinus, or salivary glands. This broad inclusion criterion ensured that a diverse range of HNC patients were represented in the study.

Exclusion criteria included patients who had undergone treatment for thyroid cancer or had any other benign neoplasm-causing trismus.

The primary outcome of our study was to determine the prevalence of trismus in HNC patients at a tertiary cancer center. Data were collected from the electronic medical record, which includes variables such as demographic details, tumor area, radiological assessment, treatment received, and mouth opening measurements.

Statistical analysis of the collected data was performed using Microsoft Excel (Microsoft® Corp., Redmond, WA) and Statistical Product and Service Solutions (SPSS, version 23; IBM SPSS Statistics for Windows, Armonk, NY) software. Frequency and percentage were used for categorical data. Bivariate analysis and chi-square tests were used to identify associations between categorical variables and the development of trismus.

## Results

Out of the 162 patients enrolled in the study, 77 (46.9%) patients have trismus. One hundred twenty-five (77.2%) were male, and the majority of the study participants were aged between 41 and 65 years. Approximately 83 (51.2%) of the participants were non-smokers, and 92 (56.8%) refrained from alcohol consumption. The most common diagnosis among the study population was oral carcinoma of the oral cavity, accounting for 80 (49.7%) of the cases. The average measured mouth opening is 36.1 mm, with a minimum mouth opening of 7 mm and a maximum of 69 mm (Table 1).

**Table 1:** Tumor characteristics of the study population T - Size of the tumor, N - Number of lymph nodes, M - Metastasis, T1 - Tumor 2 cm or less in greatest dimension, T2 - Tumor more than 2 cm but not more than 4 cm in greatest dimension, T3 - Tumor more than 4 cm in greatest dimension, T4 - Tumor invades adjacent structure, N0 - No regional lymph node metastasis, N1 - Metastasis in a single ipsilateral lymph node of 3 cm or less in greatest dimension, N2 - Metastasis in single or multiple ipsilateral lymph nodes more than 3 cm but less than 6 cm, N3 - Metastasis in lymph nodes more than 6 cm in dimension, M0 - No distant metastasis

Characteristics of the study population		n	%
Gender	Female	37	22.8%
	Male	125	77.2%
Age in years	Below 40	7	4.32%
	41 to 65	88	54.32%
	66 above	51	31.48%
	Not reported	16	9.87%
Marital status	Not married	8	4.9%
	Married	76	46.9%
	Not reported	78	48.1%
Smoking	Never smoked	83	51.2%
	Daily smoker	25	15.4%
	Former daily smoker	45	27.8%
	Not reported	9	5.6%
Smokeless tobacco	Absent	119	73.5%
	Present	29	17.9%
	Not reported	14	8.6%
Alcohol consumption	Abstainer	92	56.8%
	Low consumption	36	22.2%
	Moderate consumption	12	7.4%
	High consumption	6	3.7%
	Not reported	16	9.9%
Tumor area	carcinoma of lip	4	2.5%
	Carcinoma of the oral cavity	80	49.7%
	Oropharyngeal carcinoma	22	13.58%
	Nasopharyngeal carcinoma	6	3.7%
	Hypopharyngeal carcinoma	4	2.5%
	Carcinoma in larynx	22	13.58%
	Carcinoma in salivary glands	1	0.6%
	Not reported	23	14.19%
T	T1	50	30.9%
	T2	38	23.5%
	T3	23	14.2%
	T4	22	13.6%
	Not reported	29	17.9%
N	N0	85	52.5%
	N1	6	3.7%
	N2	32	19.8%
	N3	10	6.2%
	Not reported	29	17.9%
M	M0	128	79.0%
	Not reported	34	21.0%
Mouth opening (in mm)		36.11±14.7	-

Among the 162 patients diagnosed with HNC, most cases of carcinoma reported were squamous cell carcinoma (90.1%) and did not involve the bones or muscles. Approximately 103 (63.6%) of the patients underwent surgery, specifically wide local excision combined with selective neck dissection. Reconstruction using a soft tissue flap was performed in 43 (26.5%) of cases. Additionally, 43 patients received adjuvant chemoradiotherapy, with image-guided radiotherapy (IGRT) being the most common radiotherapy technique used (Table 2).

**Table 2:** Tumor involvement and diagnostic characteristics of the study participants ITF - Infra-temporal fossa, WLE - Wide local excision, SND - Selective neck dissection, DRT - Definitive radiotherapy, IMRT - Intensity-modulated radiotherapy, IGRT - Image-guided radiotherapy, VMAT - Volumetric modulated arc radiotherapy

Tumor involvement and diagnostic characteristics			n	%
Type of oral cancer	Squamous cell carcinoma		146	90.1
	Verrucous carcinoma		6	3.7
	Mucoepidermoid carcinoma		2	1.2
	Adenoid cystic carcinoma		4	2.5
	Basal cell adenoma		2	1.2
	Amelanotic melanoma		1	0.6
	Pleomorphic adenoma		1	0.6
Bone clinico radiologic assessment		No involvement	108	66.7%
		Yes and abutting	9	5.6%
		Yes, through and through cortical erosion	15	9.3%
		Not reported	30	18.5%
Bone involvement		No bony involvement	107	66.0%
		Mandible anterior body involvement	4	2.5%
		Mandible lateral body involvement	4	2.5%
		Mandible ramus involvement	1	0.6%
		maxilla involvement	10	6.2%
		Both maxilla and mandible involvement	2	1.2%
		Not reported	34	21.0%
CT-ITF involvement		Present	59	36.6%
		Absent	42	26.1%
		Not reported	60	37.3%
Muscle involved		None	52	32.1%
		Masseter	3	1.9%
		Temporalis	4	2.5%
		Not reported	103	63.6%
Last treatment		No treatment done	7	4.3%
		Surgery	103	63.6%
		Radiotherapy	42	25.9%
		Not reported	10	6.2%
Surgical characteristics		Lip split	3	1.9%
		Per oral	11	6.8%
		Pull through	3	1.9%
		WLE	11	6.8%
		WLE AND SND	35	21.6%
		Not reported	99	61.1%
Lip split		Angle split	45	27.8%
		No lip split	39	24.1%
		Upper lip	1	0.6%
		Not reported	77	47.5%
Lip/ commissure split		No resection of lip or commissure	98	60.5%
		Not reported	64	39.5%
Max/mand reconstruction		No resection	73	45.1%
		Only mandible	1	0.6%
		Only maxilla	12	7.4%
		Both mandible and maxilla	3	1.9%
		Marginal mandibulectomy	7	4.3%
		Segmental mandibulectomy	11	6.8%
		Not reported	55	34.0%
Flap reconstruction		No need for reconstruction	32	19.8%
		Bone free flap	7	4.3%
		Regional pedicled flap	14	8.6%
		Split skin graft	1	0.6%
		Free flap-soft tissue flap	43	26.5%
		Not reported	65	40.1%
Adjuvant		Concurrent chemoradiotherapy	43	26.5%
		RT only	32	19.8%
		Not reported	87	53.7%
Type of radiotherapy		3DRT	6	3.7%
		IMRT	9	5.6%
		IGRT	22	13.6%
		TomoTherapy	8	4.9%
		Radical	11	6.8%
		VMAT	9	5.6%
		Not reported	97	59.9%

Sociodemographic characteristics such as gender and age group showed a significant association with the development of trismus (p < 0.05). However, factors such as smoking and alcohol consumption did not show a significant association (Table 3).

**Table 3:** Presence of trismus based on sociodemographic characteristics

Characteristics of the study population		Trismus present	Trismus absent	Total	P value
Gender	Female	23(30.26)	14(16.28)	37	0.027
	Male	53(69.74)	72(83.72)	125	
	Total	76	86	162	
Age group (in years)	Less than or equal to 40	16(21.1)	7(8.14)	23	0.038
	41 to 60	31(40.79)	34(39.53)	65	
	Greater than 61	29(38.16)	45(52.32)	74	
	Total	76	86	162	
Smoking	Never smoked	42(61.76)	41(48.23)	83	0.098
	Daily smoker	12(17.64)	13(15.29)	25	
	Former daily smoker	14(20.58)	31(36.47)	45	
	Total	68	85	153	
Smokeless tobacco	Absent	48(73.85)	71(85.54)	119	0.059
	Present	17(26.15)	12(14.46)	29	
	Total	65	83	148	
Alcohol consumption	Abstainer	42(65.64)	50(60.97)	92	0.347
	Low consumption	14(21.87)	22(26.83)	36	
	Moderate consumption	7(10.93)	5(6.1)	12	
	High consumption	1(1.56)	5(6.1)	6	
	Total	64	82	146	

Regarding tumor characteristics and their association with trismus development, tumor area, and bone involvement showed a significant association (p < 0.05), but factors such as the size of the tumor (T) and the number of lymph nodes (N) did not show a significant association (Table 4).

**Table 4:** Presence of trismus according to tumor characteristics T - Size of the tumor, N - Number of lymph nodes

Tumor characteristics		Trismus present	Trismus absent	Total	P value
Tumor area	Carcinoma of lip	2(3.2)	2(2.63)	4	0.004
	Carcinoma of the oral cavity	45(71.43)	35(46.05)	80	
	Oropharyngeal carcinoma	8(12.69)	14(18.42)	22	
	Nasopharyngeal carcinoma	4(6.34)	2(2.63)	6	
	Hypopharyngeal carcinoma	1(1.59)	3(3.95)	4	
	Carcinoma of larynx	2(3.17)	20(26.32)	22	
	Carcinoma of salivary glands	1(1.59)	0(0)	1	
	Total	63	7	139	
Bone clinico-radiologic assessment	No involvement	46(73.02)	62(89.86)	108	0.022
	Yes and abutting	5(7.94)	4(5.79)	9	
	Yes, through and through cortical erosion	12(19.04)	3(4.35)	15	
	Total	63	69	132	
T	T1	16(34.8	24(38.1	40(36.7	0.511
	T2	13(28.3	21(33.3	34(31.2	
	T3	7(15.2	11(17.5	18(16.5	
	T4	10(21.7	7(11.1	17(15.6	
	Total	46	63	109	
N	N0	27(58.7)	42(66.7)	69(63.3)	0.671
	N1	2(4.3)	3(4.8)	5(4.6)	
	N2	15(32.6)	14(22.2)	29(26.6)	
	N3	2(4.3)	4(6.3)	6(5.5)	
	Total	46	63	109	

## Discussion

Trismus, characterized by restricted mouth opening, is a common complication in HNC patients, significantly impacting their quality of life. Normal mouth opening is in the range of 36-55 mm, with ≤ 35 mm indicating trismus [8]. Trismus can lead to difficulties in nutrition [10], communication, and oral hygiene maintenance, affecting an overall health-related quality of life (HRQoL) over time [10].

Various factors contribute to trismus in HNC patients, including tumor infiltration into the muscles of mastication and temporomandibular joint, radiation-induced fibrosis, and post-surgical scarring [7,11]. The prevalence of trismus in treated HNC patients varies widely in the literature, ranging from 5% to 79% [12-16]. This variation can be attributed to differences in evaluation criteria, follow-up periods, inclusion criteria, and ethnic variations in the study populations.

In this study, 77 (46.9%) of the participants presented with trismus according to Dijkstra's criteria [8]. However, caution should be exercised in interpreting this value, as the tools used to define trismus may not be validated for the specific population involved.

The association between age and trismus could be established in our study analogous to what is observed by van der Geer et al. [16], and this is contradictory to other studies [17,18]. Patients become weaker with age, ensuing increased vulnerability, declined adaptive capacity, and delayed recovery periods after treatment, augmenting the risk of trismus even further [19,20]. The range of motion also diminishes due to the micro and macro changes of joints and ligaments impairing the functions of the temporomandibular joint.

The current study suggests a significant correlation between the tumor location and the development of trismus (p < 0.05) (Table 4). The data show an increased prevalence of oral and oropharyngeal carcinomas. These findings can be comparable to the conclusions of earlier studies [15,21-24] stating that tumors located near the temporomandibular joint and masticatory muscles have increased the risk of trismus. This may be attributed to tumor infiltration or fibrosis near these structures limiting the jaw movements and inducing trismus [15,24,25]. The study also proposes tumor involvement in the bones as a potential indicator for trismus. The data from earlier studies also support this finding [16]. Our study could not establish any association between trismus and the type or size of the tumor, TNM grading, or metastasis. This finding contradicts earlier studies and can be attributed to the limited sample size and the incongruity in the designated sample population [13,26].

Marking the factors linked to trismus allows clinicians to take precautionary measures [7,21] that would reduce the risk of trismus for future patients. Patients at risk also benefitted through the early warning to tackle the situation with the preventive exercises. Literature supports preventive/early starting of exercise therapy and compliance of the patient as major factors in the management of trismus. Public health initiatives should emphasize the importance of routine screening for trismus in high-risk populations, early intervention, and patient education about preventive exercises. Compliance with exercise therapy is crucial for the successful management of trismus, underscoring the need for programs that support patient adherence to recommended therapies.

Clinical implications

Trismus is a well-known, persistent challenge commonly associated with the treatment and recovery phases in head and neck oncology. It tends to persist over time and may fluctuate in severity. The initial six months are more crucial with more fluctuations in maximum inter-incisal opening, and our study indicates an enhanced risk for some subgroups. Early intervention has proven beneficial in the prevention and rehabilitation of trismus. Implementing protocols that include device-based jaw exercises, caregiver reminders, and strong social support can positively influence outcomes. This study has identified that nearly one-half of the patients with HNC develop trismus and recognized the factors associated with trismus for the considered population. This helps the clinicians in adopting the preventive measures in time and limit the consequences.

Limitations and future directions

A potential limitation of this study is its retrospective design, which may introduce biases due to the reliance on previously collected data. The study was conducted at a single tertiary hospital, which may not reflect the prevalence and factors associated with trismus in other regions or healthcare settings. Our study did not assess outcomes such as speech or any other complications affecting the quality of life.

This study included a diverse group of HNC patients, encompassing various patient, tumor, and treatment characteristics. The identified risk factors associated with trismus need to be studied in a longitudinal fashion in a larger sample size. This will help develop a model for risk score assessment in the specific population and predict trismus based on arbitrarily chosen characteristics. While Dijkstra’s criteria are widely accepted, they have not been validated for the specific population studied here. Adapting these criteria to account for the community’s unique nutritional and cultural factors is essential for more accurate predictions of trismus.

## Conclusions

Nearly half of HNC patients develop trismus. Factors such as gender, age, use of smokeless tobacco, and tumor characteristics - particularly the tumor area and bone involvement - are significantly associated with the development of trismus. From a public health perspective, understanding these risk factors is essential for developing targeted interventions and preventive measures. Implementing routine screening for trismus in at-risk populations, along with early and tailored management strategies, can significantly improve patient outcomes and quality of life. Public health initiatives should also focus on education and awareness campaigns about the risks of smokeless tobacco and the importance of early detection and treatment of HNC. By addressing these factors, healthcare providers can enhance the overall effectiveness of cancer care and support for affected patients.
